# Secretion of proteins and antibody fragments from transiently transfected endothelial progenitor cells

**DOI:** 10.1111/jcmm.15511

**Published:** 2020-07-01

**Authors:** Loree Heller, Reynald Thinard, Melanie Chevalier, Sezgi Arpag, Yu Jing, Ruth Greferath, Richard Heller, Claude Nicolau

**Affiliations:** ^1^ Department of Medical Engineering University of South Florida Tampa Florida USA; ^2^ ALSaTECH Tufts Biolabs Launchpad Boston Massachusetts USA; ^3^ Center for Bioelectrics Old Dominion University Norfolk Virginia USA; ^4^ Friedman School of Nutrition Science and Policy Tufts University Boston Massachusetts USA; ^5^ Present address: Department of Physiological Sciences Eastern Virginia Medical School Norfolk Virginia USA

**Keywords:** electroporation, endothelial progenitor cells, ex vivo cell therapy, gene therapy, β‐amyloid disaggregation

## Abstract

In neurodegenerative diseases such as Alzheimer's disease, Parkinson's disease, multiple sclerosis and amyotrophic lateral sclerosis, neuroinflammation can lead to blood‐brain barrier (BBB) breakdown. After intravenous or intra‐arterial injection into mice, endothelial progenitor cells (EPCs) home to the damaged BBB to promote neurovascular repair. Autologous EPCs transfected to express specific therapeutic proteins offer an innovative therapeutic option. Here, we demonstrate that EPC transfection by electroporation with plasmids encoding the reporter protein GFP or an anti‐β‐amyloid antibody fragment (Fab) leads to secretion of each protein. We also demonstrate the secreted anti‐β‐amyloid Fab protein functions in β‐amyloid aggregate solubilization.

## 1 INTRODUCTION

The blood‐brain barrier (BBB) consists primarily of endothelial cells with pericytes, astrocytes and microglia on a basement membrane ^1^, ^2^ and functions to regulate the transport of cells and proteins into the normal brain. Approximately 98% of therapeutic agents are excluded by this barrier. In several conditions, including neurodegenerative diseases such as Alzheimer's disease (AD), Parkinson's disease, multiple sclerosis and amyotrophic lateral sclerosis (ALS), neuroinflammation can lead to BBB breakdown and leakiness. Endothelial progenitor cells (EPCs) migrate to the damaged BBB in response to hypoxia,^3^ trauma, growth factor ^4^, ^5^, ^6^, ^7^ or chemokine secretion.^8^ Integration of the EPCs into the compromised BBB promotes neurovascular repair.^9^, ^10^, ^11^, ^12^ EPCs can also be transfected ex vivo to produce therapeutic molecules such as antibody fragments (Fabs) directed against misfolded proteins for neuroprotection.

At least 26 clinical trials are registered on www.clinicaltrials.gov using EPCs as therapeutic agents for indications such as ischaemic heart disease, pulmonary arterial therapy and decompensated liver cirrhosis.^13^ Autologous EPCs transfected to express specific therapeutic proteins offer an option to treat these and other indications. Transfection can be performed using a number of modalities including several viruses, which can produce long‐term expression. Non‐viral vectors such as plasmids can be delivered into cells by mechanical, chemical or physical means, primarily producing transient expression since genomic integration does not occur. Electroporation, a physical method, was used in this study because this technique does not require external reagents that may generate unintended consequences.

Anti‐β‐amyloid monoclonal antibodies (mAbs) for treatment of AD have been tested in several major Phase III clinical trials. Such antibodies against different epitopes within the biomarker Aβ_1‐42_ yielded excellent results in vitro and in transgenic mice.^14^, ^15^, ^16^, ^17^, ^18^, ^19^, ^20^ Phase I and II clinical trials by AC Immune,^21^ Hoffmann‐La Roche,^16^, ^22^, ^23^ Eli Lilly ^17^, ^23^, ^24^ and Biogen ^19^, ^25^, ^26^ have demonstrated encouraging results. However, despite differences in the nature of these antibodies, each failed in Phase III, although later some therapeutic activity was demonstrated with aducanumab (Biogen). The cause(s) of these failures have not been clearly elucidated. Possible causes may be either an increasing lack of therapeutic significance of β‐amyloid, which is very unlikely if we take into account the in vitro and in vivo preclinical results, or the fact that the mAbs do not reach their target, the β‐amyloid deposits, in a sufficient amount. This is likely due to the strong filtering effect of the BBB. High doses of injected antibodies (60 mg per kg body weight dose) were necessary with aducanumab (Biogen) to obtain a modest effect.^19^

We report here a novel targeted delivery system consisting of ex vivo transfected, autologous endothelial precursor cells (EPCs) capable of homing to the BBB and expressing therapeutic Fabs. These cells were obtained for the development of targeted cell‐mediated gene therapy to a hypoxic site.^27^ EPCs are able to repair the damaged BBB and blood‐spinal cord barrier characteristic of neurodegenerative diseases such as AD,^28^ ALS,^29^, ^30^ traumatic brain injury ^31^, ^32^ or stroke ^33^, ^34^ and will thus have a double function when combined with Fab production.

## 2 MATERIALS AND METHODS

### 2.1 Cells

hCMEC/D3 blood‐brain barrier endothelial cells, isolated from human temporal lobe microvessels and immortalized by lentiviral vector transduction with the catalytic subunit of human telomerase (hTERT) and SV40 large T antigen, were grown in Endogro MV complete medium (EMD Millipore, Temecula, CA). HEPC.CB1 and MAgEC10.5, human and murine endothelial progenitor cell lines, respectively, immortalized by retroviral transduction encoding hTERT,^35^, ^36^, ^37^ kind gifts of C. Kieda, were grown in Optimem containing 2% FBS (Gibco, Thermo Fisher Scientific) at 37°C with 5% CO_2_.

### 2.2 Plasmids

Commercially available reporter plasmids encode firefly luciferase (gWizLuc) or enhanced green fluorescent protein (GFP, gWizGFP), both driven by the CMV promoter plus intron (Aldevron). pSF‐CAG.InsSP‐GFP encodes the insulin secretion sequence in frame with enhanced GFP driven by the synthetic CAG (CMV enhancer, chicken beta‐actin promoter and rabbit beta‐globin splice acceptor site) promoter. To create pSF‐CAG.InsSP‐GFP, the OG4678 vector (OxGene), encoding the CAG promoter, was used as parent vector. PCR was performed to append the human insulin signal peptide to EGFP. Restriction and ligation were performed with the EGFP PCR product and OG4678 to create the final construct for expression and secretion of EGFP. pl.DualCAG.Hygro.cAb2789 was created with pSF‐CAG.InsSP‐GFP as parent vector and encodes both chains of an anti‐β‐amyloid Fab with optimized peptide signals driven by dual CAG promoters. A His Tag is included to simplify the screening of the Fab production. The parent vector was restricted and subsequently ligated with a restricted DNA fragment corresponding to the ubiquitin promoter, a downstream hygromycin resistance marker for cell selection and a polyadenylation sequence. PCR was performed to append optimized peptide signals to both chains of the anti‐β‐amyloid Fab‐encoding sequences (C_H1_‐V_H_ and C_L_‐V_L_). In addition, a 10‐His Tag encoding sequence was added to the heavy chain encoding sequence. Finally, the fragments were subcloned into the parent vector with CAG promoters for both chains to create the final vector expressing and secreting the anti‐β‐amyloid Fab. pSF‐CAG.InsSP‐GFP and pl.DualCAG.Hygro.cAb2789 were verified with restriction digests and Sanger sequencing. All plasmids were commercially prepared with endotoxin levels confirm to be <100 EU/mg (Aldevron; OxGene) and diluted to 2 mg/mL in physiological saline.

### 2.3 Transfection

Electroporation was performed in cuvettes using an ECM 830 (BTX Harvard Apparatus). For simplicity, cells were suspended in culture medium throughout the delivery. The tested pulse protocols were chosen from the literature (Table 1).

**Table 1:** Electroporation protocols

Name	Pulse protocol	Reference
PR0329	1p 900 V/cm^a^ 20 ms	BTX protocol database, personal Communication, Dr Kurt Engleka, Thomas Jefferson University
PR0462	1p 625 V/cm 5 ms	^43^
2pPR0462	2p 625 V/cm 5 ms 1 Hz^b^	–
‘M’	7p 411 V/cm 0.1 ms 1 Hz	^39^

^a^ Volts/centimetre.

^b^ hertz.

### 2.4 Reporter assays

Luciferase activity was quantified 20 hours after transfection in medium containing 250 µg/mL luciferin using an Omegastar or Clariostar microplate reader (BMG Labtech). Intracellular GFP was quantified by flow cytometry (MACSQuant Analyzer 10, Miltenyi Biotec, Bergisch Gladbach, Germany) and FlowJo analysis software (BD Biosciences). In brief, single HEPC.CB1 EPCs were gated out using the doublets discrimination analysis technique based on FCS and/or SSC scatter plots. GPF‐positive cells among all singlets were then identified and analysed for their frequency and median fluorescent intensity (MFI). Secreted GFP in medium was quantified using a FLUOstar Omega or Clariostar microplate reader (BMG Labtech).

### 2.5 Viability assays

PrestoBlue (Invitrogen, Thermo Fisher Scientific) in medium was added 20 hours after transfection. After two‐hour incubation, reagent reduction was quantified using a FLUOstar Omega or Clariostar microplate reader (BMG Labtech).

### 2.6 PCR

Total RNA was extracted from EPCs (PureLink RNA Micro kit; Invitrogen) according to the manufacturer's instructions. Complementary DNA was obtained by reverse transcription (SuperScript II Reverse Transcriptase, Invitrogen). Custom primers Forward 5’CTC CAA CTA CTG GAT GAA CTG GGT GAA G3’ and Reverse 5’CCT CGC TGG TCA GAG AGC TCA3’ (Amplification length: 182bp, Thermo Fisher Scientific) were validated with dilutions of pl.DualCAG.Hygro.cAb2789 (100% efficiency, data not shown) and absolute quantification of anti‐β‐amyloid Fab mRNA was performed using real‐time quantitative PCR with Power Up SYBR Green Master mix (Applied Biosystems) on a QuantStudio 6 (Thermo Fisher Scientific).

### 2.7 Microscopy

MAgEC 10.5 EPCs were resuspended, counted with Luna cell counting slides (Logos Biosystems, Gyeonggi‐do, South Korea) and transfected with pDNA as previously described. The cells were then transferred to CorningTM PrimariaTM 24‐well plates (Thermo Fisher Scientific) and cultured for 48 hours at 37°C 5% CO2. The cells were fixed with 4% formaldehyde in PBS for 30 minutes, permeabilized with 0.5% Triton X‐100 in PBS for 20 minutes, washed three times in PBS and blocked with 3% bovine serum albumin in PBS for 1 hour at room temperature.

Cells were stained with a chimeric human/rabbit anti‐His Tag antibody (Sigma‐Aldrich; cat# SAB5600096) at a dilution of 1:500 in 3% BSA in PBS overnight at 4°C, washed three times in PBS and then incubated with Goat Anti‐Human IgG antibody (Fc specific)−FITC antibody (Sigma‐Aldrich; cat# F9512) at a dilution 1:100 and DAPI at a dilution of 1/100 (Millipore), both prepared in 3% BSA in PBS for 1 hour at room temperature. The PBS was removed and two more washes were performed before analysis for the emission of fluorescence with a digital microscope (CELENA S, Logos Biosystems). Alternatively, cells were stained with a rabbit anti‐His Tag antibody (Abcam; cat# ab232492) at a dilution of 1:100 in 3% BSA in PBS overnight at 4°C, washed three times in PBS and then incubated with Goat Anti‐rabbit IgG − AlexaFluor 488 antibody (Abcam; cat#150077) at a dilution 1:1000 and DAPI at a dilution of 1/100 (Millipore), both prepared in 3% BSA in PBS for 1 hour at room temperature and processed as described above.

### 2.8 Solubilization capacity of β‐amyloid aggregates

Reaction tubes containing 30 µg of β‐amyloid peptide 1‐42 (Bachem) in 10 µL of PBS, pH 7.4 (Gibco), were incubated for 1 week at 37°C. Aggregation was measured by the thioflavin T (ThT)‐binding assay, in which the dye's fluorescence emission intensity reflects the degree of fibrillar aggregation. Disaggregation was followed after addition of concentrated cell supernatant, purified antibodies to the preformed fibres (10 µL each) or an irrelevant control antibody. The supernatant was concentrated 100‐fold using Amicon Ultra 30 K columns (Millipore) with centrifugation 30 minutes at 3900 g. The purified Fab and irrelevant control antibody (mouse IgG) were used at a final concentration of 1.5 mg/mL. The reaction incubated for 2 days at 37°C. Fluorescence (excitation: 450 nm; emission: 482 nm) was measured after addition of 1 mL of ThT (3 µM in 50 mM sodium phosphate buffer, pH 6.0) on Fluoromax4C fluorometer (Horiba).

### 2.9 Statistics

The statistical significance between the groups was determined by analysis of variance with Tukey‐Kramer multiple comparisons test (GraphPad Software) or by Student's *t* test. A *P* value <.05 was considered significant.

## 3 RESULTS

**Figure 1:**
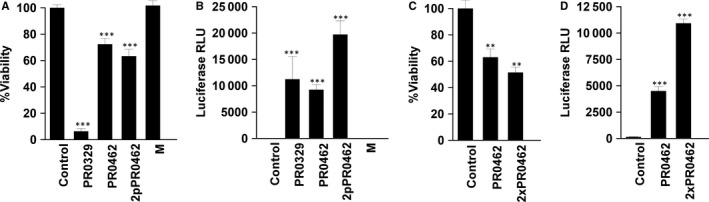
Development of transfection method for human endothelial cells. A, HEPC.CB1 EPC viability after luciferase plasmid delivery as determined by reducing potential; B, HEPC.CB1 EPC luciferase reporter expression; n = 3‐9 independent experiments. C, mature endothelial cell viability with protocol modification; D, mature endothelial cell luciferase reporter expression with protocol modification. RLU, relative luminescence units, n = 3. **P < .01; ***P < .001 statistical difference from control group. Mean ± SEM

Since the autologous EPCs necessary for ex vivo gene therapies may be isolated in limited numbers, particular attention was paid to maintaining cell viability. With the exception of protocol ‘M’, all of the pulse protocols tested (Table 1) significantly affected EPC cell viability (Figure 1A) to varying degrees. Transgene expression after luciferase plasmid delivery was then compared (Figure 1B). While protocol PR0329 produced significant reporter expression, only 6% of cells survived pulse delivery. A significantly (*P* < .001) greater percentage of cells survived protocol PR0462, and a similar level of transgene expression was detected. Since this was a single pulse protocol, we performed parallel testing with two pulses (2pPR0462). This had the effect of increasing expression twofold while having no additional effect on viability. Finally, while protocol ‘M’ had no significant effect on viability, no reporter expression was observed. The mature BBB cell line hCMEC/D3 was also tested using these conditions. In these cells, pulsing with one or two pulses similarly decreased viability (Figure 1C). In this case, luciferase activity increased nearly threefold with the two‐pulse protocol, a rate similar to that of EPCs (Figure 1D). Subsequent experiments were performed using the two‐pulse version of PR0462.

**Figure 2:**
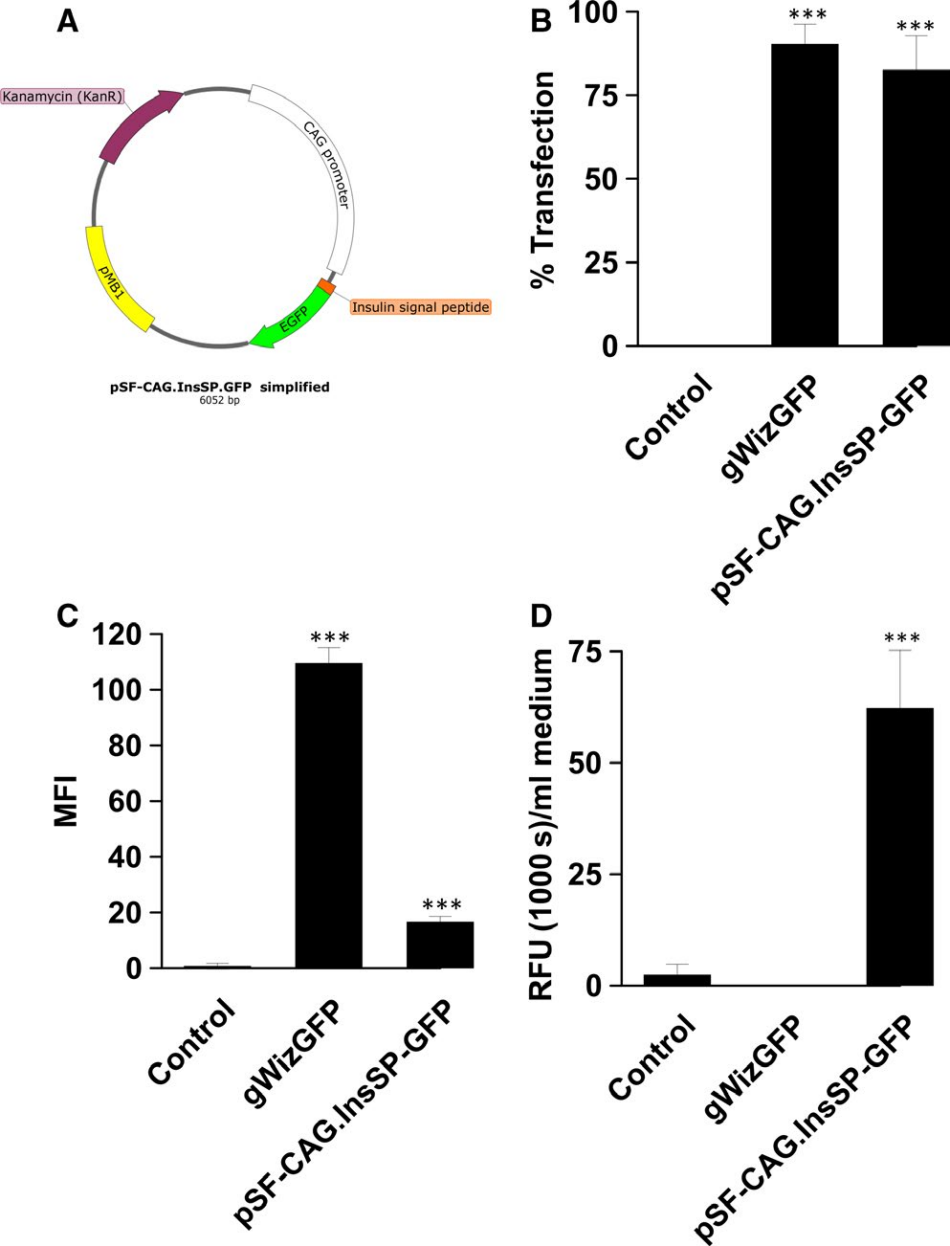
Transfection efficiency and confirmation of GFP secretion by HEPC.CB1 EPCs. A, pSF‐CAG.InsSP‐GFP map B, Flow cytometry of gWizGFP and pSF‐CAG.InsSP‐GFP transfected cells; C, median fluorescent intensity (MFI); D, secretion of GFP into medium. RFU, relative fluorescence units. ***P < .001 statistical difference from control group

A plasmid encoding GFP was designed to generate reporter protein secretion as a model for soluble protein therapies (pSF‐CAG.InsSP‐GFP, Figure 2A). Transfection efficiency and median cell fluorescence were quantified by flow cytometry and compared between this plasmid and a commercially available GFP plasmid (gWizGFP). Transfection efficiency did not vary significantly between the two plasmids, 82.6% ± 4.2% and 90.4% ± 2.4%, respectively (Figure 2B). However, GFP secretion was reflected in the median fluorescent intensity (MFI), where the MFI of cells transfected with gWizGFP was 6.6‐fold (*P* < .001) higher than that of cells transfected with pSF‐CAG.InsSP‐GFP (Figure 2C). Secretion was confirmed by quantifying GFP in the medium (Figure 2D). GFP was not detected in the medium after gWizGFP transfection, while significant GFP levels were detected in the medium of EPCs transfected with pSF‐CAG.InsSP‐GFP.

**Figure 3:**
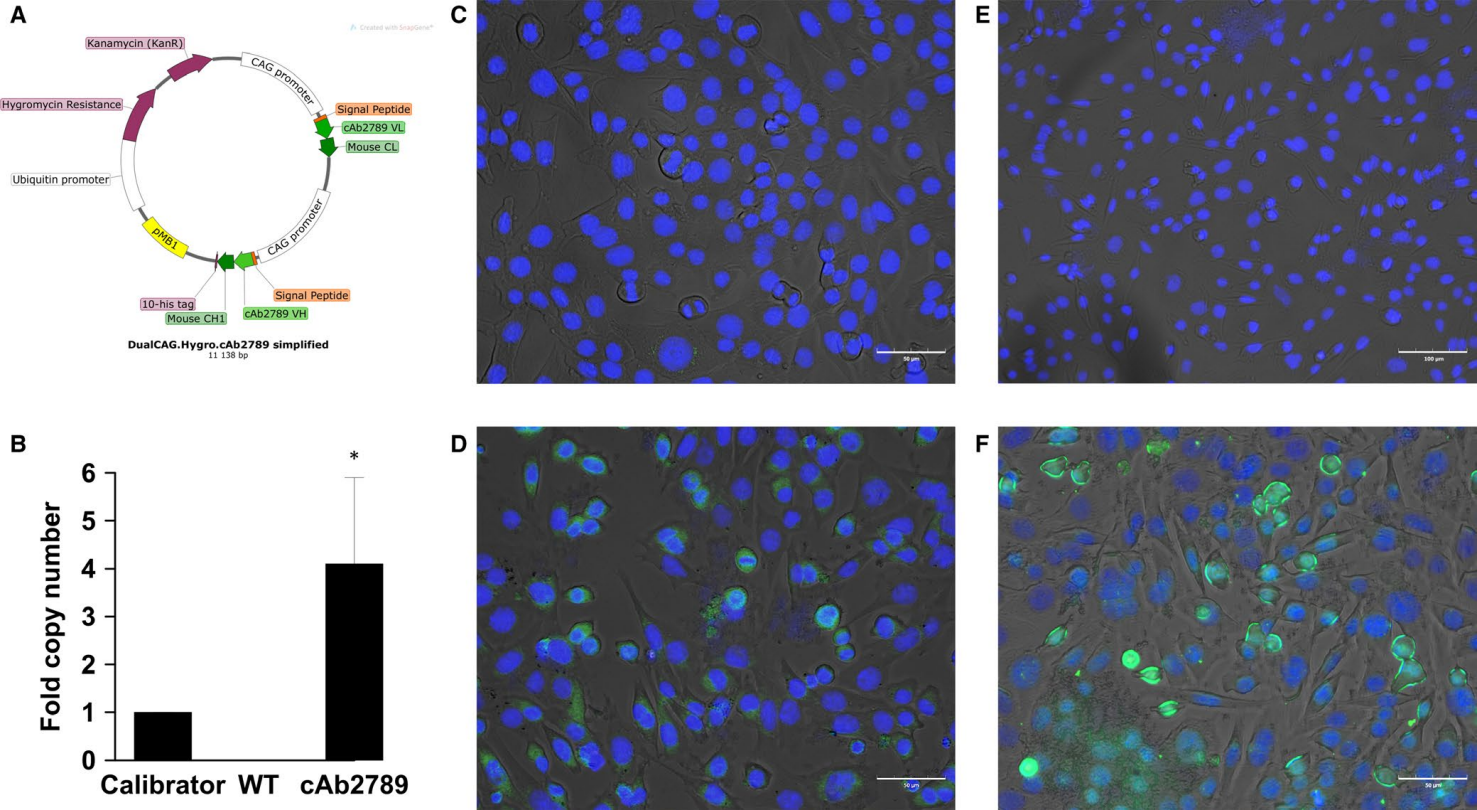
Confirmation of antigen binding fragment production by MAgEC 10.5 EPCs. A, pl.DualCAG.Hygro.cAb2789 map, B, Reverse transcription qPCR of the cAb2789 in transfected cells, n = 3, *P < .05 statistical difference from wild type. Representative images of complete staining of C, non‐transfected cells with chimeric anti‐His Tag antibody, D, cells transfected with plasmid pl.DualCAG.Hygro.cAb2789 with chimeric anti‐His Tag antibody, E, non‐transfected cells with rabbit anti‐His Tag antibody, F, cells transfected with plasmid pl.DualCAG.Hygro.cAb2789 with rabbit anti‐His Tag antibody. His Tag is shown in green; cell nuclei stained with DAPI are shown in blue

Fab‐encoding plasmids were designed and MAgEC 10.5 EPCs were transfected to test the ability of transfected EPCs to produce and secrete Fabs (pl.CAG. cAb2789, Figure 3A). Transgene mRNA was detected in the cells 48 hours after transfection (Figure 3B). These levels were higher after delivery of the single dual plasmid than after delivery of the combination of two plasmids (data not shown).

The presence of anti‐β‐amyloid Fab protein was confirmed microscopically 48 hours after transfection. Primary antibody controls and pSF‐CAG.InsSP‐GFP transfected control cells did not show staining (data not shown). Non‐transfected negative controls (Figure 3C, 3E) also did not show significant staining. Expression was detected in cells transfected with pl.DualCAG.Hygro.cAb2789 using both a chimeric anti‐His Tag antibody (Figure 3D) and a rabbit anti‐His Tag antibody (Figure 3F). Therefore, both mRNA and protein assays confirmed the expression of the transfected Fab.

**Figure 4:**
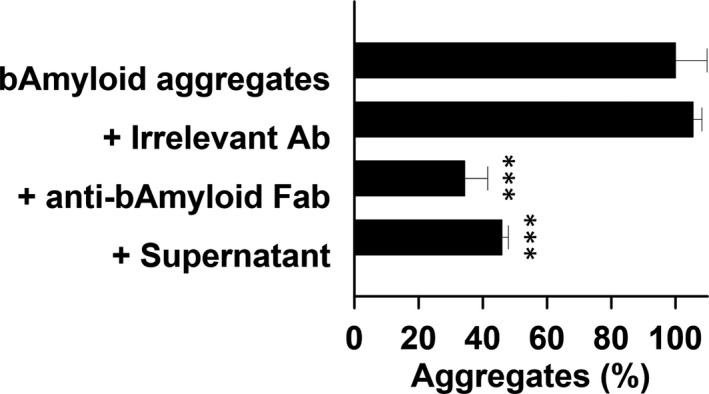
Solubilization of β‐Amyloid aggregates by concentrated supernatant from transfected MAgEC 10.5 EPCs, n = 3. Aggregates we incubated with an irrelevant antibody, purified anti‐β‐amyloid Fab protein, or with concentrated supernatant. ***P < .001 statistical difference from aggregates alone

An aggregate solubilization assay was used to demonstrate both secretion and function of the anti‐β‐amyloid Fab protein (Figure 4). Incubation of an irrelevant antibody had no effect on the aggregates. A significant reduction in aggregates was observed after incubation with either an anti‐β‐amyloid Fab or concentrated medium from transfected cells.

## 4 DISCUSSION

In this study, we demonstrated that EPCs can be transfected using electroporation at high efficiency with a minor loss of cell viability. Transfected EPCs are capable of secreting a reporter protein. Finally, we show that transfected EPCs can also secrete functional antibody fragments.

We first tested HEPC.CB1 EPC transfection using publicly described pulse protocols. In the early development of electroporation as a delivery technique, an exponential pulse shape, essentially a capacitor discharge, was used for cell transfection. This pulse shape was previously tested in EPCs ^38^ compared with viral delivery and was found to be unsuitable. In this study, we chose square wave pulse protocols to better control cell exposure to electric pulses. While delivery with pulse protocol PR0329, originally developed for human umbilical vein endothelial cells (HUVECs), produced significant luciferase expression, it also massively reduced cell viability. HUVEC viability was not assayed in the original PR0329 protocol. The reverse was true with protocol ‘M’,^39^ which did not produce detectable cell killing; neither did it produce detectable luciferase expression. It must be noted that protocol ‘M’ was developed for drug rather than for plasmid delivery to human microvascular endothelial cells. A pulse protocol similar to PR0462 was previously described.^40^ HUVECs were suspended in a specific buffer formulation with plasmid and exposed 5ms pulses at a frequency of 1 Hz. While the voltage‐to‐distance ratio was similar (600 vs. 625 V/cm), a larger number of pulses (8) were delivered. The maximum transfection efficiency observed was 40% with an approximately 10% viability determined using microscopic morphology. Delivery with protocol PR0462 produced some loss of cell viability, but also significant luciferase reporter expression. This single pulse protocol was developed for delivery to HUVECs, but increasing to two pulses increased luciferase levels approximately twofold while maintaining cell viability. This comparison was made in BBB endothelial cells with similar results. With two pulses, no additional effect on viability was observed, but luciferase levels increased 2.4‐fold. This implies that this pulse protocol can reproducibly deliver pDNA across related cell types.

Transfection efficiency was quantified using two different plasmids encoding GFP, a classic reporter plasmid and a plasmid specifically designed for GFP secretion. A transfection efficiency of greater than 80% was observed in HEPC.CB1 EPCs using either of these plasmids. As expected, intracellular GFP was significantly lower in the plasmid designed for secretion than the classic plasmid, while significant levels of GFP were detected in the cell medium.

We next confirmed production and function of secreted anti‐β‐amyloid Fab after transfection. Production of Fab mRNA was confirmed via qPCR while production of the Fab protein was confirmed microscopically. Function of the secreted anti‐β‐amyloid Fab was demonstrated using an aggregate solubilization assay. These results support the therapeutic potential of transfected EPCs.

For clinical application of this technology, long‐term expression may be desirable for therapeutic efficacy. In that case, viral delivery, particularly with adeno‐associated virus (AAV) is unquestionably a promising transfection option. Although AAV integrates preferentially into chromosome 19, random integration may produce insertional mutagenesis.^41^ Non‐integrating AAV vectors will avoid this problem; however, transgene expression is diluted with cell replication.^42^

The purpose of the present study was the demonstration that early EPCs transfected ex vivo with an Fab‐encoding plasmid would express and secrete functional Fabs that could solubilize β‐amyloid aggregates. The work reported here constitutes the first step toward a complex in vivo study aiming to demonstrate the insertion of injected EPCs into the mouse BBB without damage to the BBB, the penetration of the transfected EPCs and the secretion of the expressed Fabs into the brain parenchyma. This work is underway.

## CONFLICTS OF INTEREST

RT, MC, RG and CN are shareholders and employees of ALSaTECH Inc, Boston, MA, USA, and have a patent application relevant to the subject covered in this manuscript. LH and RH are consultants to ALSaTECH Inc, Boston, MA, USA. SA and YH have no conflict of interest to declare.

## AUTHOR CONTRIBUTION

**Loree Heller:** Formal analysis (equal); Investigation (equal); Methodology (equal); Validation (equal); Writing‐original draft (equal); Writing‐review & editing (equal). **Reynald Thinard:** Formal analysis (equal); Investigation (equal); Methodology (equal); Validation (equal); Writing‐review & editing (equal). **Mélanie Chevalier:** Investigation (equal); Methodology (equal). **Sezgi Arpag:** Investigation (equal); Methodology (equal). **Yu Jing:** Investigation (equal); Methodology (equal). **Ruth Greferath:** Methodology (equal); Resources (equal). **Richard Heller:** Investigation (equal); Writing‐review & editing (equal). **Claude Nicolau:** Conceptualization (equal); Methodology (equal); Project administration (equal); Resources (equal); Validation (equal); Writing‐review & editing (equal).

## Data Availability

The data that support the findings of this study are available from the corresponding authors upon reasonable request.
